# Reduced mitochondrial translation prevents diet-induced metabolic dysfunction but not inflammation

**DOI:** 10.18632/aging.104010

**Published:** 2020-10-06

**Authors:** Kara L. Perks, Nicola Ferreira, Judith A. Ermer, Danielle L. Rudler, Tara R. Richman, Giulia Rossetti, Vance B. Matthews, Natalie C. Ward, Oliver Rackham, Aleksandra Filipovska

**Affiliations:** 1 Harry Perkins Institute of Medical Research, Centre for Medical Research, QEII Medical Centre, University of Western Australia, Nedlands, Western Australia, Australia; 2 School of Pharmacy and Biomedical Sciences, Curtin University, Bentley, Western Australia, Australia; 3 School of Biomedical Sciences, University of Western Australia, Perth, Western Australia, Australia; 4 Medical School, Royal Perth Hospital Unit, University of Western Australia, Perth, Western Australia, Australia; 5 School of Public Health and Curtin Health Innovation Research Institute, Curtin University, Perth, Western Australia, Australia; 6 Curtin Health Innovation Research Institute, Curtin University, Bentley, Western Australia, Australia; 7 School of Molecular Sciences, The University of Western Australia, Crawley, Western Australia, Australia

**Keywords:** mitochondria, stress response, mTOR and insulin signaling pathways, obesity, metabolic syndrome

## Abstract

The contribution of dysregulated mitochondrial gene expression and consequent imbalance in biogenesis is not well understood in metabolic disorders such as insulin resistance and obesity. The ribosomal RNA maturation protein PTCD1 is essential for mitochondrial protein synthesis and its reduction causes adult-onset obesity and liver steatosis. We used haploinsufficient *Ptcd1* mice fed normal or high fat diets to understand how changes in mitochondrial biogenesis can lead to metabolic dysfunction. We show that Akt-stimulated reduction in lipid content and upregulation of mitochondrial biogenesis effectively protected mice with reduced mitochondrial protein synthesis from excessive weight gain on a high fat diet, resulting in improved glucose and insulin tolerance and reduced lipid accumulation in the liver. However, inflammation of the white adipose tissue and early signs of fibrosis in skeletal muscle, as a consequence of reduced protein synthesis, were exacerbated with the high fat diet. We identify that reduced mitochondrial protein synthesis and OXPHOS biogenesis can be recovered in a tissue-specific manner via Akt-mediated increase in insulin sensitivity and transcriptional activation of the mitochondrial stress response.

## INTRODUCTION

Mitochondria have fundamental regulatory roles in metabolism, from the breakdown of lipids and carbohydrates for energy production, synthesis of fats and hormones, to cell signaling via metabolites and reactive oxygen species [1, 2]. Antegrade signaling from the nucleus or cytoplasm to mitochondria can modulate energy levels and biogenesis of the respiratory chain and is becoming well understood. Nutrient deprivation or energy consuming stresses can lead to upregulation of the mTOR signaling pathway that can stimulate energy production by increasing mitochondrial biogenesis [3, 4]. In contrast, retrograde signaling, from mitochondria to the rest of the cell, is not as well understood. Mitochondrial dysfunction can have varied effects on cellular gene activation and protein synthesis depending on the severity of the insult. Severely reduced oxidative phosphorylation (OXPHOS) can signal via AMPK as a stress response to low ATP levels. In addition, increased carbon one metabolism [5–8] and activation of ATF4 and ATF5 are common stress responses to severe OXPHOS impairments that can be caused by mutations or loss of proteins involved in mitochondrial biogenesis and OXPHOS function [9, 10]. Cellular responses to low or moderate changes in mitochondrial function have only recently started to be explored [11], to understand how these may be mediated or how they may be rescued by the cell. Mutations in *C. elegans* genes encoding mitochondrial ribosomal proteins have been shown to have positive effects on mitochondrial function by priming OXPHOS function, leading to increased lifespan [12, 13].

Mitochondrial gene expression is essential for OXPHOS biogenesis and energy production and we have shown that severely impaired regulation of the mitochondrial genome [14, 15] or protein synthesis [16] leads to premature death that cannot be compensated by increasing mTOR signaling and one carbon metabolism [16]. Gene expression in mammalian mitochondria is predominantly regulated by RNA-binding proteins (RBPs) [17] whose roles have been intimately linked to energy production as well as carbohydrate and lipid metabolism [2, 18, 19]. A significant number of mitochondrial RBPs characterized to date are essential for life, whereas haploinsufficiency of most RBPs is sufficient for normal development. Recently, we identified that the RBP pentaricopeptide repeat domain protein 1 (PTCD1), that is required for 16S rRNA stability, pseudouridylation and mitoribosome assembly [16], when in a heterozygous state can cause metabolic imbalance in aged mice [20]. We found that moderate changes in mitochondrial protein synthesis and OXPHOS biogenesis in these mice perturb metabolism and cause adult onset obesity [20].

We have used this unique model to investigate how reduced mitochondrial protein synthesis can affect energy metabolism in response to different energy demands. Here we show that reduced mitochondrial protein synthesis can protect against metabolic syndrome early in life before adult-onset obesity develops in mice fed a normal chow diet (NCD). Protein synthesis can be recovered on a high fat diet (HFD) specifically in the liver of mice via Akt-mediated increased insulin sensitivity in the liver but not in skeletal muscle, where high fat diet leads to inflammation in response to reduced insulin responsiveness.

## RESULTS

### Young *Ptcd1^+/-^* mice are resistant to high fat diet-induced weight gain and have increased glucose tolerance and insulin sensitivity

**Figure 1:**
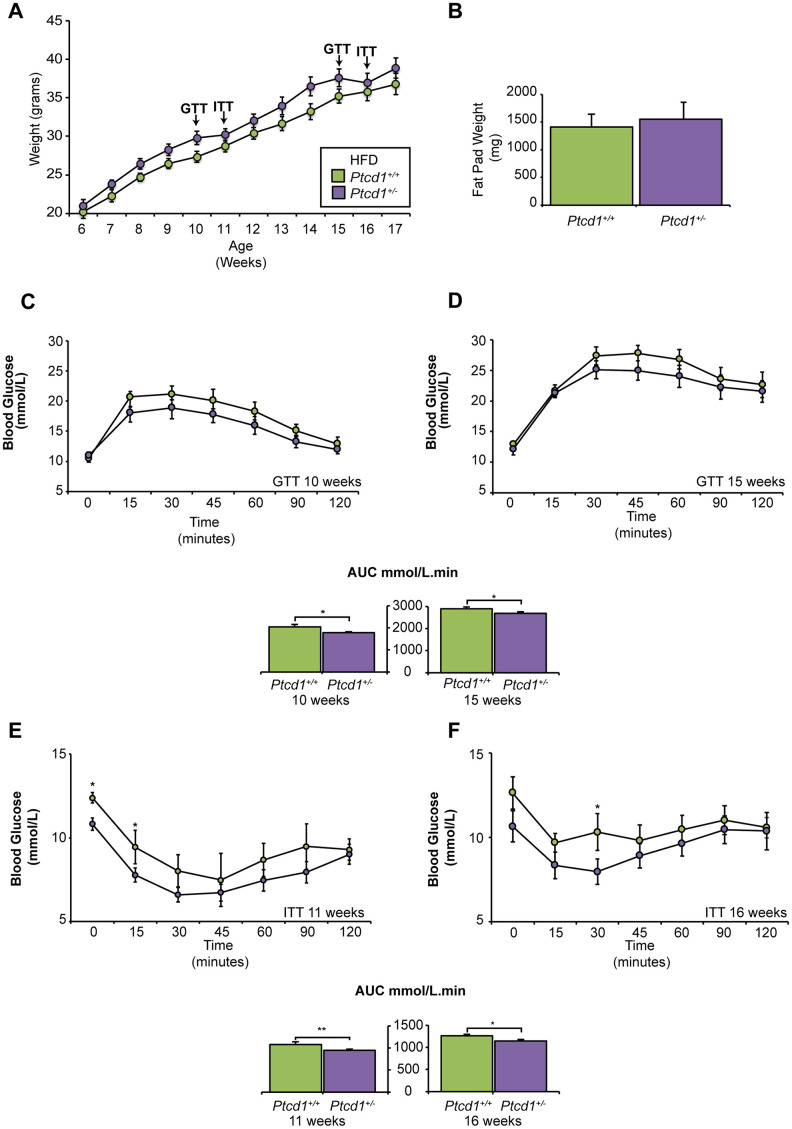
**The effects of high fat diet on mice with reduced mitochondrial protein synthesis.** (**A**) Weight gain in grams from 12 weeks of HFD feeding between *Ptcd1^+/+^* (n=10) and *Ptcd1*^+/-^ (n=10) mice (**B**) Weight of intra-abdominal epididymal fat pads in grams for 17-week-old *Ptcd1*^+/+^ (n=5) and *Ptcd1*^+/-^ (n=5) mice fed a HFD. (**C, D**) Glucose tolerance in 10- and 15-week-old *Ptcd1*^+/+^ (n=10) and *Ptcd1*^+/-^ (n=10) mice. Quantitative values are area under the curve (AUC) ± SEM. *P < 0.05, Student’s t test. (**E, F**) Insulin sensitivity in 11- and 16-week-old *Ptcd1*^+/+^ (n=10) and *Ptcd1*^+/-^ (n=10) mice. Quantitative values are area under the curve (AUC) ± SEM. *P < 0.05, Student’s t test.

Recently we showed that reduced mitochondrial protein synthesis caused by haploinsufficiency of PTCD1 led to increased weight gain in haploinsufficient mice (*Ptcd1^+/-^*) on a NCD from 15 weeks of age compared to their wild type littermates (*Ptcd1^+/+^*), causing adult onset obesity by 30 weeks of age [20]. To understand the mechanisms causing adult onset obesity we investigated the metabolic changes that occur in these mice from an early age, (6 to 17 weeks old) and the effects of HFD during this period. There was no significant difference in weight from week 6 to week 14 between *Ptcd1*^+/+^ and *Ptcd1^+/-^* mice on a NCD, but from 15 weeks, the *Ptcd1*^+/−^ mice showed a significant increase in weight compared to controls (Supplementary Figure 1A) and as we have previously reported [20]. The HFD led to an increased trend in the weight of the *Ptcd1^+/-^* mice relative to control mice, however, this was not significant (Figure 1A) and the *Ptcd1^+/-^* mice did not have different food intake relative to control mice over the 12-week HFD challenge (Supplementary Figure 2A). Furthermore, there was no difference in the intra-abdominal epididymal pad weights in the *Ptcd1^+/-^* mice compared to controls at 17 weeks of age in mice fed either diet (Figure 1B and Supplementary Figure 1B) and the expression level of *Ptcd1* was ~50% in the *Ptcd1^+/-^* mice fed on either NCD or HFD (Supplementary Figure 2B).

*Ptcd1*^+/−^ mice fed a NCD were glucose-intolerant at 15 weeks of age (Supplementary Figure 1C, 1D) [20]. In contrast, glucose tolerance testing (GTT) of mice fed a HFD showed that the *Ptcd1^+/-^* mice were more glucose tolerant at both 10 and 15 weeks of age (Figure 1C, 1D). Insulin tolerance testing (ITT) revealed, NCD fed *Ptcd1*^+/−^ mice were slightly more insulin-sensitive at 11 weeks of age; but, by 16 weeks of age, there were no changes in insulin sensitivity between *Ptcd1*^+/+^ and *Ptcd1*^+/−^ NCD mice (Supplementary Figure 1E–1F and [20]). Interestingly, ITT revealed that at 11 weeks of age the HFD *Ptcd1^+/-^* mice were more insulin sensitive compared to control mice (Figure 1E). Furthermore, at 16 weeks of age *Ptcd1^+/-^* mice were more insulin sensitive and had greater glucose lowering efficacy compared to their littermate controls (Figure 1F), suggesting that reduction in mitochondrial protein synthesis, measured in liver and skeletal muscle mitochondria on both diets (Supplementary Figure 3A–3D), may improve insulin sensitivity early in life.

### High fat diet stabilizes hormonal changes associated with metabolic syndrome, but does not protect from inflammation

**Figure 2:**
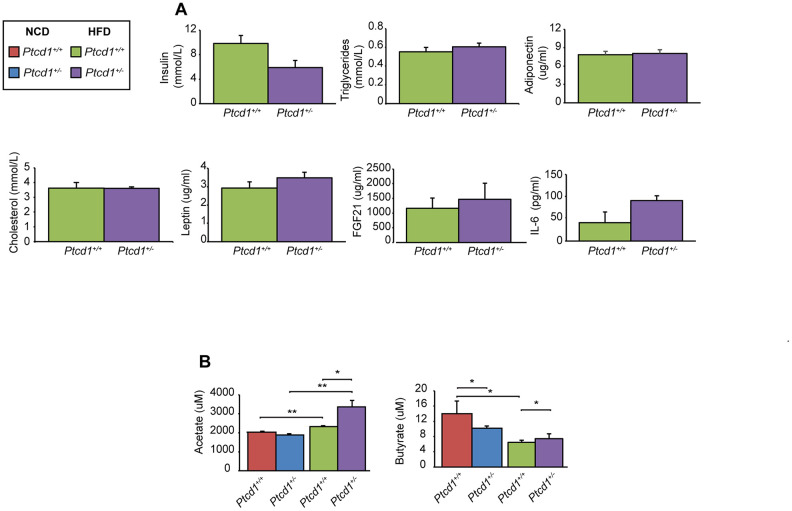
**Hormonal and microbiome changes in response to reduced mitochondrial protein synthesis in mice fed a normal and high fat diet.** (**A**) Insulin, triglycerides, adiponectin, cholesterol, leptin, FGF21 and IL-6 levels were measured in serum obtained from 17-week-old *Ptcd1*^+/+^ (n=5) and *Ptcd1*^+/-^ (n=5) mice after 12 weeks of HFD feeding. (**B**) The short chain fatty acids butyrate and acetate were measured in serum collected from *Ptcd1*^+/+^ (n=5) and *Ptcd1*^+/-^ (n=5) mice. Error bars are SEM. *P < 0.05, **P<0.01, Student’s t test.

Next, we investigated the effects of HFD on the *Ptcd1^+/+^* and *Ptcd1^+/-^* mice by measuring circulating insulin, triacylglycerides, hormones and growth factors involved in glucose, lipid metabolism, regulation of food intake and energy expenditure by enzyme-linked immunosorbent assays (ELISAs) at the end of the 12-week HFD challenge. In *Ptcd1^+/-^* mice, the levels of insulin, triglycerides, cholesterol, adiponectin and leptin were not significantly different, between the control and haploinsufficient mice (Figure 2A), consistent with the absence of weight differences between these mice. This indicates that the HFD may be beneficial, since on a NCD the *Ptcd1^+/-^* mice leptin levels were significantly elevated [20]. Similarly, FGF-21, was increased in mice on NCD as a marker of mitochondrial dysfunction [20], whereas on a HFD there were no differences in the FGF-21 levels between the *Ptcd1^+/+^* and *Ptcd1^+/-^* mice, further indicating that reduced mitochondrial protein synthesis protects against the effects of a HFD (Figure 2A).

In aged *Ptcd1^+/-^* mice circulating IL-6 levels were significantly increased [20], which was consistent with chronic liver injury and activation of the c-Jun N-terminal kinase (JNK) inflammatory pathway. Therefore, we measured circulating IL-6 serum levels as well as signaling IL-6 from tissue homogenates from the white adipose tissue (WAT), skeletal muscle and liver of NCD and HFD fed young mice. Circulating levels of IL-6 were unchanged in the *Ptcd1*^+/-^ mice compared to controls when fed a HFD (Figure 2A). Furthermore, on a NCD the signaling levels of IL-6 were not changed in any of the tissues from the *Ptcd1^+/+^* and *Ptcd1^+/-^* mice (Supplementary Figure 4), indicating that decreased protein synthesis and HFD do not affect IL-6 levels early in life.

Short chain fatty acids (SCFA) produced by the gut microbiota have been identified as markers of metabolic change. Butyrate and acetate are abundant SCFAs in the colon and increased levels of these SCFAs have been shown to protect from diet induced obesity and metabolic disorders [21, 22]. Therefore, we measured the levels of circulating SCFA in serum of *Ptcd1^+/+^* and *Ptcd1^+/-^* mice fed either NCD or HFD (Figure 2B). Acetate levels were significantly increased in serum of HFD *Ptcd1^+/-^* mice but not in NCD-fed *Ptcd1^+/-^* mice compared to controls (Figure 2B), consistent with the previously reported protective role of acetate in diet induced obesity [22–26] and the HFD protective effects in *Ptcd1^+/-^* mice. Butyrate levels were decreased in *Ptcd1^+/-^* mice fed on a NCD, whereas the HFD resulted in increased levels of butyrate in the *Ptcd1^+/-^* mice compared to their respective control mice (Figure 2B). The butyrate levels were reduced in the *Ptcd1^+/+^* mice on the HFD compared to the NCD but not in the *Ptcd1^+/-^* mice (Figure 2B), indicating that decreased mitochondrial protein synthesis may protect against high fat diet induced metabolic changes.

### Lipid accumulation is reduced in the livers of *Ptcd1*^+/-^ mice on a HFD and a HFD causes inflammation of the WAT and increased skeletal muscle fibrosis

**Figure 3:**
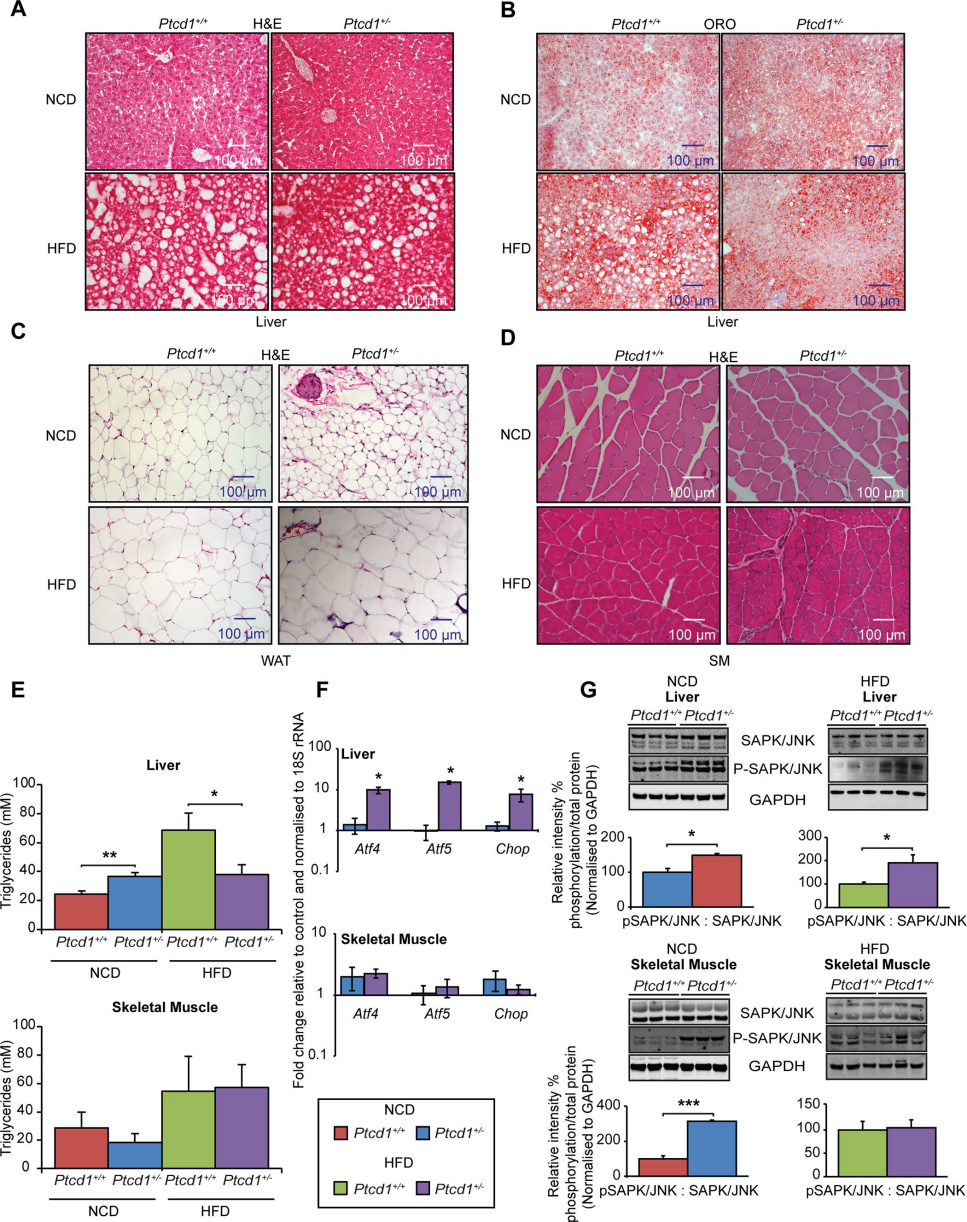
**High fat diet can specifically protect the liver from impaired mitochondrial biogenesis by upregulating a transcriptional stress response.** (**A**) Liver sections cut to 10-μm thickness were stained with H&E, from NCD or HFD fed *Ptcd1*^+/+^ (n=5) and *Ptcd1*^+/-^ (n=5) mice. (**B**) Liver sections cut to 10 μm thickness were stained with Oil red O and hematoxylin, from NCD or HFD fed 17-week-old *Ptcd1*^+/+^ (n=5) and *Ptcd1*^+/-^ (n=5) mice. (**C**) White adipose tissue sections cut to 5-μm thickness were stained with H&E, from 17-week-old *Ptcd1*^+/+^ (n=5) and *Ptcd1*^+/-^ (n=5) mice fed either a NCD or HFD. (**D**) Skeletal muscle tissue sections cut to 5-μm thickness were stained with H&E, from *Ptcd1*^+/+^ (n=5) and *Ptcd1*^+/-^ (n=5) mice fed either a NCD or HFD. Scale bars are 100 μm. (**E**) Triacylglycerol levels were measured in total liver and skeletal muscle isolated from NCD and HFD *Ptcd1^+/+^* (n=9) and *Ptcd1^+/-^* (n=9) mice. Mitochondrial transcription factor mRNAs *Atf4*, *Atf5* and *Chop* were measured in total liver (**F**) and skeletal muscle RNA isolated from NCD and HFD *Ptcd1^+/+^* (n=5) and *Ptcd1^+/-^* (n=5) mice by qRT-PCR and normalized to 18S rRNA. (**G**) Endogenous levels of SAPK/JNK and its phosphorylated form (Thr^183^/Tyr^185^) were determined by immunoblotting of whole tissue lysates from skeletal muscle and livers of 17-week-old *Ptcd1*^+/+^ (n=5) and *Ptcd1*^+/-^ mice (n=5) fed a NCD or HFD. GAPDH (glyceraldehyde-3-phosphate dehydrogenase) was used as a loading control. Relative protein levels and relative intensity percentage of phosphorylation versus total protein levels were measured using ImageJ software and normalized to GAPDH. Error bars are SEM. *P < 0.05, ***P<0.001, Student’s t test.

We investigated the effects of HFD on the liver, skeletal muscle, and WAT from *Ptcd1^+/+^* and *Ptcd1^+/-^* mice fed either a NCD or HFD by histology. Hemotoxylin and eosin (H&E) staining of NCD fed young *Ptcd1^+/-^* mice revealed no significant changes in the livers compared to the control littermates (Figure 3A). However, Oil Red O staining revealed higher lipid accumulation in livers from *Ptcd1^+/-^* mice on a NCD (Figure 3B). The HFD induced higher lipid accumulation in the livers from both *Ptcd1^+/+^* and *Ptcd1^+/-^* mice compared to their NCD littermates. Interestingly, the control mice had an increased number of lipid droplets that were more widespread and had a consistent distribution throughout the liver (Figure 3B). In the livers of *Ptcd1^+/-^* mice fed a HFD the steatotic vesicles were smaller and formed clusters suggesting that reduced mitochondrial protein synthesis may protect the liver from steatosis on a HFD.

The sizes of the adipocytes in the WAT of the NCD-fed *Ptcd1^+/-^* were smaller compared to controls, indicative of adipogenesis (Figure 3C). However, HFD promoted increased inflammation of the WAT in *Ptcd1^+/-^* mice, found as accumulation of crown-like structures (CLSs) that comprise infiltrating macrophages around dying or dead adipocytes [27]. In NCD-fed mice the skeletal muscle was not affected, however, HFD induced inflammation in the skeletal muscle of *Ptcd1^+/-^* mice, but not the controls, evident from the increased necrotic fibers (Figure 3D). Our findings suggest that reduced mitochondrial protein synthesis can protect from the effects of the HFD-induced pathology in the liver but not in WAT and skeletal muscle. This was further confirmed by measuring triacylglycerols in liver and skeletal muscle where triacylglycerol levels were reduced in the livers of *Ptcd1^+/-^* mice compared to control mice fed on a HFD (Figure 3E).

### Stress activates a transcriptional response specifically in the liver but not skeletal muscle

To investigate if reduced protein synthesis and subsequent stress from the HFD was able to induce the mitochondrial stress response we measured the levels of *Atf4*, *Atf5* and *Chop* mRNAs in the liver and skeletal muscle in NCD and HFD *Ptcd1^+/+^* and *Ptcd1^+/-^* mice by qRT-PCR. On a NCD reduced protein synthesis was not sufficient to activate *Atf4*, *Atf5* and *Chop* expression, however, under additional stress of the HFD there was a significant increase in their expression specifically in the liver, but not the skeletal muscle (Figure 3F).

Mitochondrial stress can be sensed by CHOP that can, in turn, activate c-Jun and JNK2 [28], therefore, we investigated the activation of the stress activated protein kinase (SAPK)/c-Jun N-terminal kinase (JNK) by measuring the steady state and phosphorylated levels of this kinase. The phosphorylation of SAPK/JNK was increased in the skeletal muscle of *Ptcd1*^+/−^ mice fed a NCD (Figure 3G). The SAPK/JNK phosphorylation levels were also increased in the liver of *Ptcd1^+/-^* mice fed a HFD compared to controls (Figure 3G), despite the decrease in liver steatosis but consistent with the increased transcriptional activation of *Atf4*, *Atf5* and *Chop* (Figure 3E). Interestingly, the phosphorylated SAPK/JNK levels were normalized in the skeletal muscle of *Ptcd1^+/-^* mice fed a HFD compared to those fed a NCD (Figure 3F), indicating that this pathway is not activated in the skeletal muscle of *Ptcd1^+/-^* mice on a HFD.

### Tissue-specific changes of Akt signaling pathways in response to reduced mitochondrial protein synthesis

**Figure 4:**
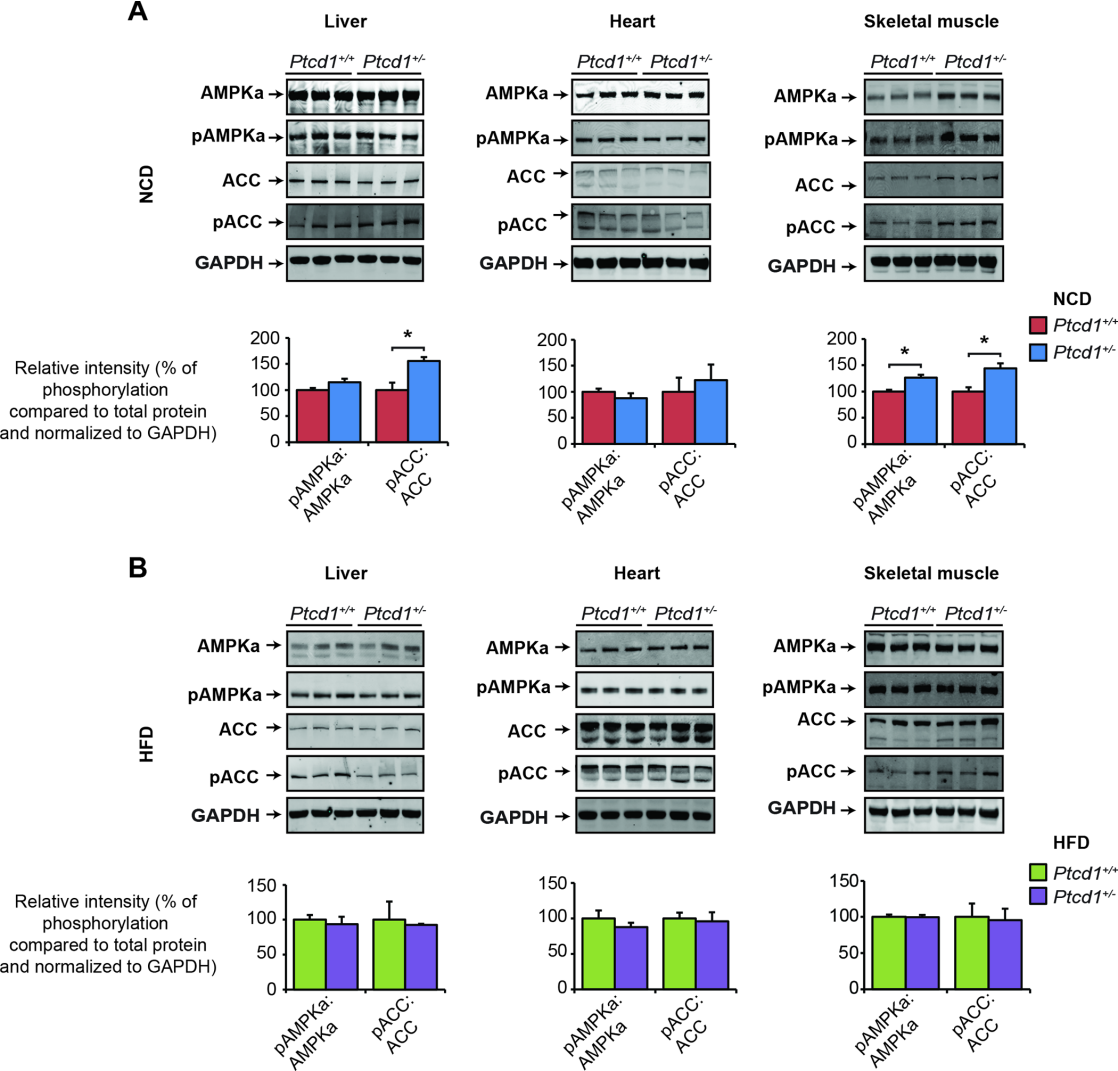
**Liver specific activation of Akt stimulates insulin sensitivity.** AMPKα and ACC signaling was measured by immunoblotting of liver, heart and skeletal muscle lysates isolated from normal chow fed (**A**) and high fat fed (**B**) *Ptcd1*^+/+^ (n=8) and *Ptcd1*^+/-^ (n=8) mice. GAPDH (glyceraldehyde-3-phosphate dehydrogenase) was used as a loading control. Relative protein levels and relative intensity of phosphorylation versus total protein levels were measured using ImageJ software and normalized to GAPDH. The data are representative of results obtained from 8 mice from each genotype. Error bars are SEM. *P < 0.05, Student’s t test.

Previously we have shown that haploinsufficiency of PTCD1 resulted in impaired insulin and fatty acid metabolism as well as differential regulation of mTOR signaling pathways [20]. Recently we also found that severe mitochondrial dysfunction causes the retrograde transcriptional upregulation of the mTOR pathway to compensate for compromised energy metabolism by upregulation of cytoplasmic protein synthesis and pro-survival genes [16]. Therefore, we investigated the effects of reduced mitochondrial protein synthesis and HFD on the regulation of several different signaling pathways. On a NCD, phosphorylated levels of ACC were increased in the livers and phosphorylated AMPKα and ACC were increased in the skeletal muscle of *Ptcd1*^+/−^ mice relative to controls (Figure 4A). These results suggest that glucose, fatty acid uptake and fatty acid oxidation may be upregulated when mitochondrial protein synthesis is decreased due to lower cellular energy. There were no significant differences in the phosphorylation levels of AMPKα and ACC on HFD in liver, heart and skeletal muscle of *Ptcd1*^+/+^ and *Ptcd1*^+/−^ mice (Figure 4B), indicating that HFD diet may improve energy production and no longer require the activation of AMPKα.

**Figure 5:**
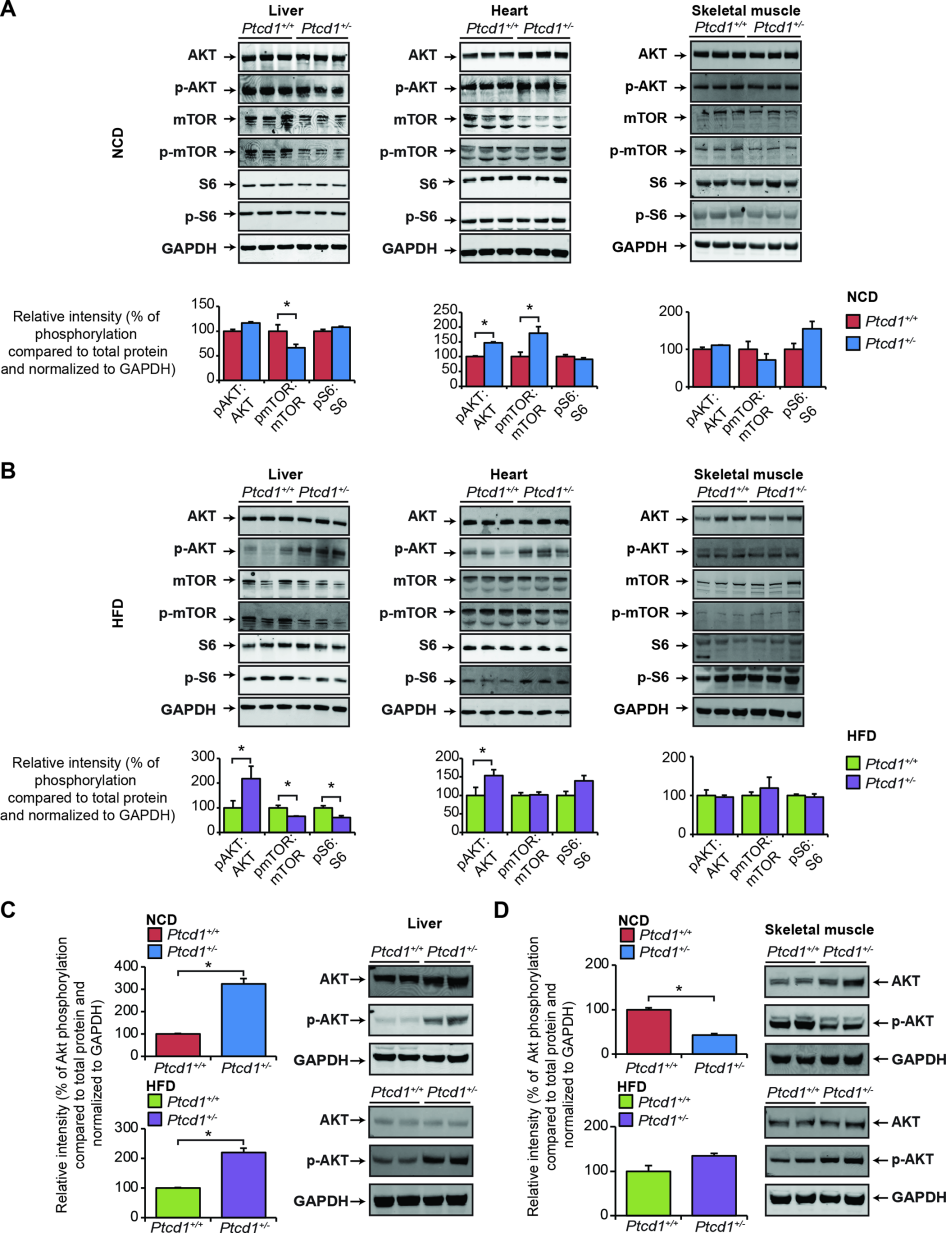
**Tissue specific regulation of the Akt and mTOR pathways in response to reduced mitochondrial protein synthesis.** Akt and mTOR signaling were measured by immunoblotting of liver, heart and skeletal muscle lysates isolated from normal chow fed (**A**) and high fat diet (**B**) *Ptcd1*^+/+^ (n=8) and *Ptcd1*^+/-^ (n=8) mice. GAPDH was used as a loading control. Relative protein levels and relative intensity of phosphorylation versus total protein levels were measured using ImageJ software and normalized to GAPDH. The data are representative of results obtained from 8 mice from each genotype. Error bars are SEM. *P < 0.05, Student’s t test. Terminal insulin testing of insulin dependent Akt activation was assessed in liver (**C**) and skeletal muscle (**D**) lysates isolated from normal chow fed and high fat diet *Ptcd1*^+/+^ (n=6) and *Ptcd1*^+/-^ (n=6) mice. Relative protein levels and relative intensity of phosphorylation versus total protein levels were measured using ImageJ software and normalized to GAPDH. Error bars are SEM. *P < 0.05, Student’s t test.

Akt is a pivotal kinase that is regulated by a number of upstream hormones, nutrients and growth factors as well as being influenced by many cellular events, including mitochondrial dysfunction [29, 30]. Akt signaling was not changed in the liver, or skeletal muscle of *Ptcd1*^+/−^ mice fed a NCD, but it was increased in the heart, likely in response to the increased steady state non-phosphorylated levels of Akt, compared to the control mice (Figure 5A). However, Akt phosphorylation was dramatically increased in the livers and hearts of *Ptcd1*^+/−^ mice fed a HFD compared to the control *Ptcd1*^+/+^ mice (Figure 5B). In contrast, the phosphorylation of Akt was unaffected in skeletal muscle of *Ptcd1*^+/−^ mice fed a HFD. (Figure 5B).

To investigate the effects of Akt in response to insulin stimulation, we performed terminal insulin testing on *Ptcd1*^+/−^ and *Ptcd1*^+/+^ mice fed either a NCD or HFD. We identified that the livers of *Ptcd1*^+/−^ mice were more insulin responsive in both NCD and HFD groups compared to their control littermates with significantly increased phosphorylated levels of Akt (Figure 5C). In the skeletal muscle of *Ptcd1*^+/−^ mice fed a NCD there was a significant decrease in the phosphorylated levels of Akt, indicating a dampened response to insulin compared to their control littermates (Figure 5D). On the HFD the phosphorylated levels of Akt normalized between the *Ptcd1*^+/−^ and control mice (Figure 5D), suggesting that HFD feeding increases skeletal muscle responsiveness to insulin via Akt signaling. Despite the Akt responsiveness to insulin it was not sufficient to protect the *Ptcd1*^+/−^ mice from skeletal muscle-specific inflammation.

Next, we investigated the mTOR signaling pathway in the livers, hearts and skeletal muscle of NCD and HFD fed *Ptcd1*^+/−^ and *Ptcd1*^+/+^ mice (Figure 5A, 5B). In the livers of NCD fed *Ptcd1*^+/−^ mice the phosphorylated levels of mTOR were significantly decreased, whereas the levels of the downstream substrate of mTOR, the ribosomal protein S6 were unaffected (Figure 5A). On a HFD, the phosphorylated levels of mTOR were significantly decreased along with the phosphorylated levels of S6 in the livers of *Ptcd1*^+/−^ mice compared to controls (Figure 5B). These changes are consistent with those identified in the livers of aged *Ptcd1*^+/−^ mice [20], indicating that when mitochondrial biogenesis is reduced during stress or ageing, the mTOR signaling is repressed in the liver to preserve mitochondrial energy levels. The phosphorylated levels of mTOR were increased in the hearts of *Ptcd1^+/-^* mice on a NCD (Figure 5A), indicating that mTOR activation is a necessary response to decreased mitochondrial function in high energy demand tissues, consistent with that identified in the hearts of aged *Ptcd1^+/-^* mice [20]. These findings validate the tissue-specific responses previously identified between the liver and heart and their respective energy requirements. The HFD likely provided the required energy for the heart to eliminate the need for mTOR activation (since non-phosphorylated and phosphorylated levels of mTOR and S6 levels were not changed) in the *Ptcd1*^+/−^ mice compared to their control littermates (Figure 5B). The lack of changes in Akt and mTOR signaling in the skeletal muscle of *Ptcd1^+/-^* mice on either diet (Figure 5A, 5B, 5D) may contribute to their susceptibility to inflammation on HFD.

mTOR is a signaling system comprised of 2 distinct protein complexes – mTOR Complex 1 (mTORC1) and mTOR Complex 2 (mTORC2) [31], where mTORC1 regulates protein synthesis and cell proliferation via S6 while mTORC2 plays a major role in Akt mediated insulin signaling and cell survival [32]. Our findings suggest that mTOR signaling acts via the mTORC2 pathway in the liver and mTORC1 in the heart as we have previously reported [20]. These findings reveal tissue-specific responses to reduced mitochondrial protein synthesis during NCD and HFD where the liver conserves energy by reducing cytoplasmic protein synthesis through downregulation of the mTOR pathway and mobilization of glucose and lipid metabolism through Akt upregulation. In contrast, in high energy demand tissues such as the heart, mitochondrial dysfunction in NCD fed mice upregulates both Akt and mTOR pathways via mTORC1 as the dependence on aerobic metabolism may require increased cytoplasmic protein synthesis to cope with the reduction in mitochondrial function.

### High fat diet causes tissue-specific changes in mitochondrial biogenesis and OXPHOS function

**Figure 6:**
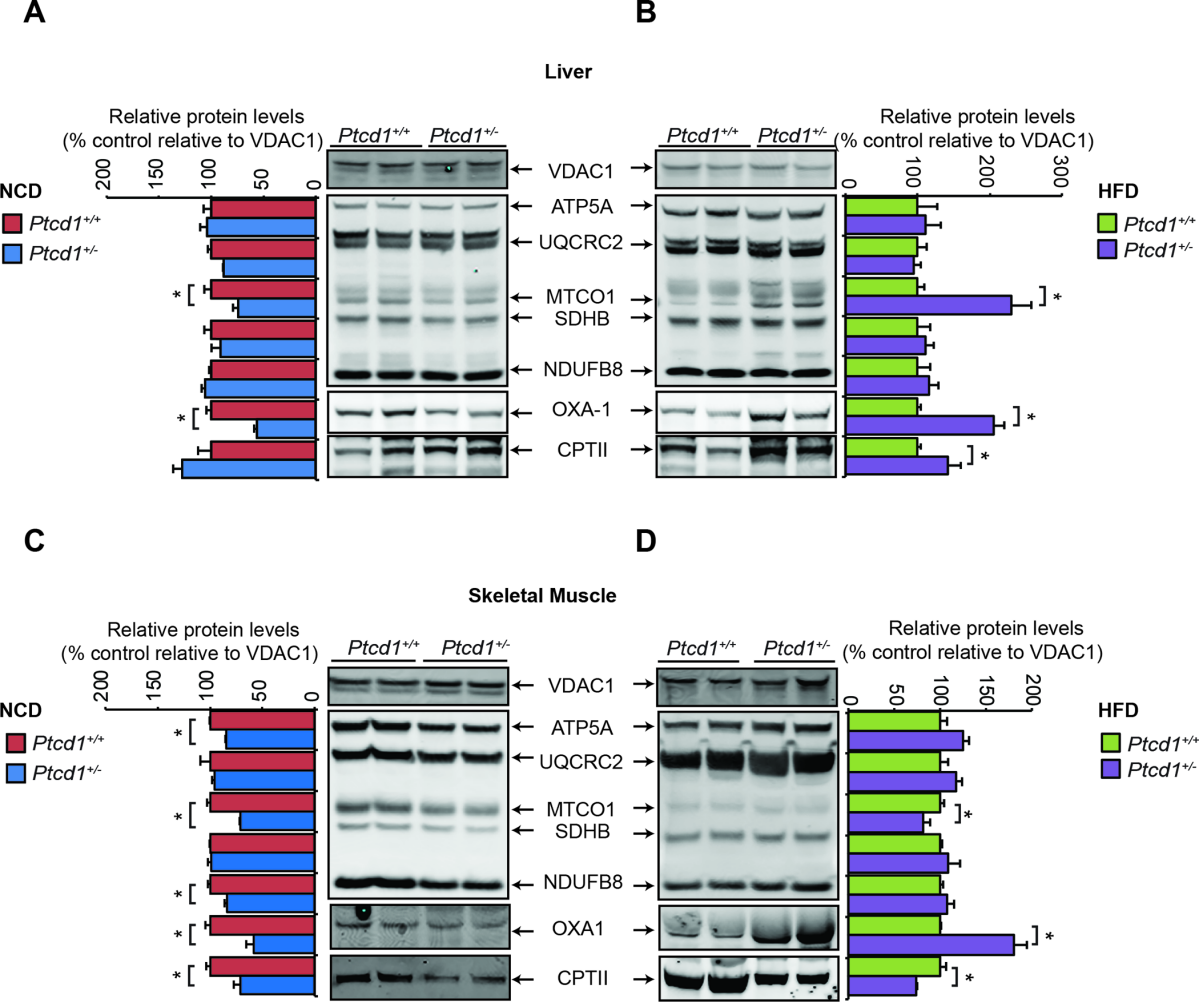
**HFD can recover mitochondrial biogenesis.** Mitochondrial proteins (50 μg) isolated from *Ptcd1*^+/+^ and two *Ptcd1*^+/-^ mice were resolved on 4-12% Bis-Tris gels and immunoblotted against antibodies to analyze the levels of nuclear and mitochondrial encoded proteins in liver (**A**, **B**) and skeletal muscle (**C**, **D**) isolated from normal chow diet (**A**, **C**) and high fat diet (**B**, **D**) fed *Ptcd1*^+/+^ (n=6) and *Ptcd1*^+/-^ (n=6) mice. VDAC1 (porin) was used as a loading control. Relative abundance of proteins was measured using ImageJ software normalized to VDAC1. Error bars indicated SEM. *P < 0.05, Student’s t test.

To investigate the effects of reduced mitochondrial protein synthesis on a NCD or HFD on mitochondrial biogenesis we immunoblotted for mitochondrial- and nuclear-encoded OXPHOS subunits. Mitochondrial biogenesis was significantly improved, and the levels of the mitochondria encoded COXI subunit were increased in the livers of *Ptcd1^+/-^* mice compared to controls on a HFD and also relative to the same mice on a NCD (Figure 6A, 6B). In skeletal muscle of *Ptcd1^+/-^* young mice fed a NCD there was a significant reduction in both the mitochondrial and nuclear encoded OXPHOS subunits (Figure 6C). In contrast to the liver, in the skeletal muscle in mice fed a HFD did not restore the steady state levels of the mitochondrially encoded COXI in the *Ptcd1^+/-^* mice (Figure 6D), indicating differential regulation of mitochondrial gene expression between these two tissues in response to stress, such as HFD. Similarly, COXI levels were not increased with the HFD in heart mitochondria of *Ptcd1^+/-^* mice (Supplementary Figure 5).

We investigated the effects on the steady-state levels of OXA1 that is required for the insertion of the mitochondrially synthesized proteins in the membrane where they reside as part of the OXPHOS complexes. Although OXA1 levels were decreased both in the livers and skeletal muscle of *Ptcd1^+/-^* mice on a NCD, reflective of the decreased protein synthesis in mitochondria, OXA1 levels were increased in both tissues of the *Ptcd1^+/-^* mice when they were fed a HFD (Figure 6B, 6D). The increase in OXA1 indicates that high fat diet could stimulate mitochondrial biogenesis to cope with reduction in translation.

To investigate if the increase in mitochondrial biogenesis is driven by the increased lipid content via fatty acid oxidation in the HFD we investigated the levels of the carnitine palmitoyltransferase II (CPTII) enzyme. CPTII along with CPTI and acylcarnitine translocase (CACT) make up the carnitine palmitoyltransferase (CPT) system that transports long chain fatty acids (LCFAs) into the mitochondrial matrix [33]. CPTII is essential for fatty acid oxidation within mitochondria, and acts by removing carnitine and adding coenzyme A to enable energy metabolism [34]. The CPTII levels were increased in the liver of HFD fed *Ptcd1^+/-^* mice compared to control mice and mice fed NCD, indicating that lipid uptake was increased in these mice (Figure 6A, 6B) consistent with their increased weight gain with age [20]. Increased CPTII levels further confirmed that higher lipid content in the diet resulted in increased lipid uptake into mitochondria and this was greater when mitochondrial protein synthesis was reduced, likely in an effort to restore energy levels. In the skeletal muscle, the levels of CPTII were decreased significantly, in *Ptcd1^+/-^* compared to *Ptcd1^+/+^* mice on both NCD and HFD (Figure 6C, 6D), indicating reduced LCFA import in the skeletal muscle.

### Maximal respiratory capacity can support mitochondrial function when protein synthesis is reduced

**Figure 7:**
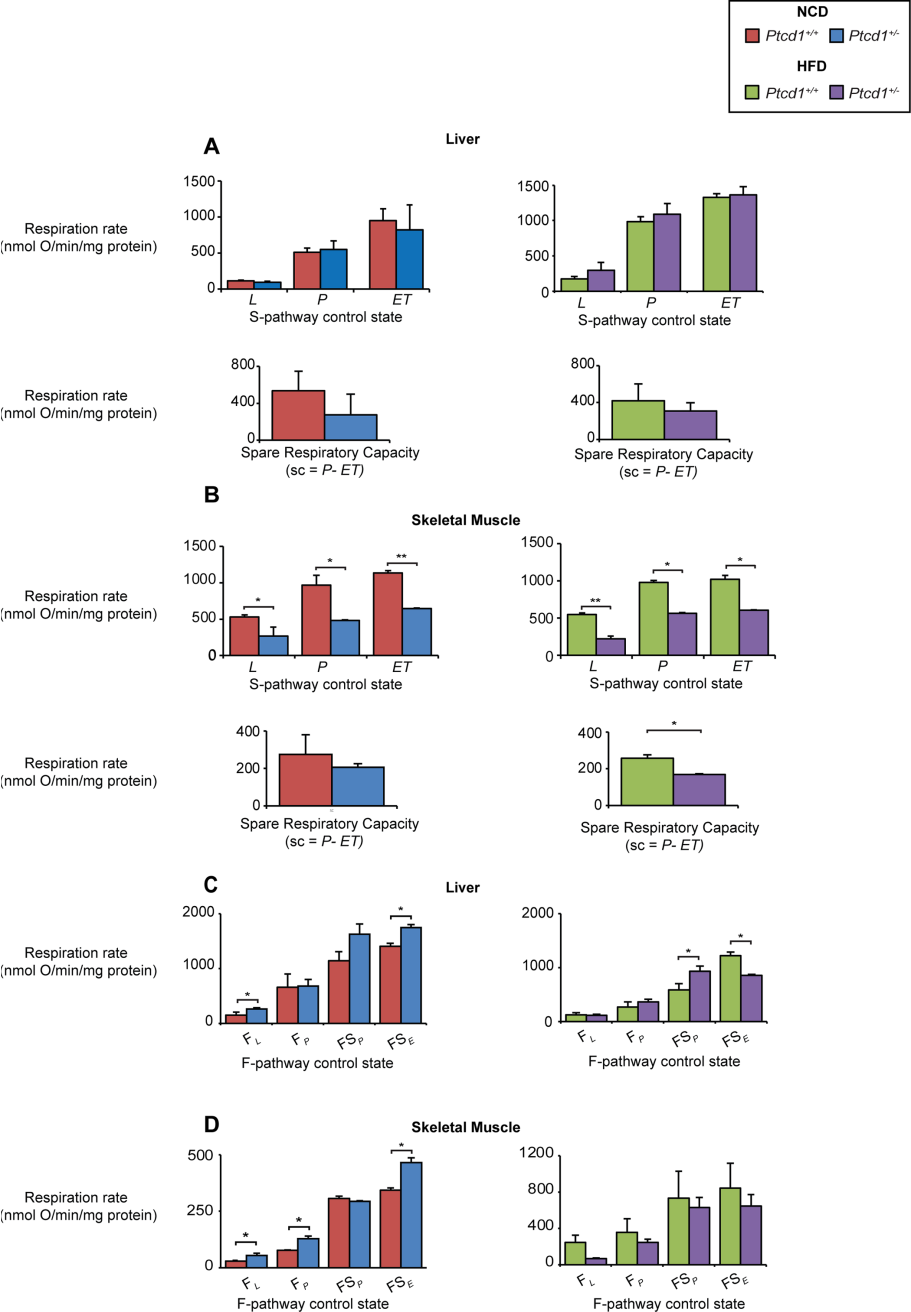
**Increase in fatty acid oxidation and respiratory capacity can compensate for reduced mitochondrial biogenesis in the liver but not skeletal muscle.** Oxygen consumption for Leak State (*L*), OXPHOS capacity (*P*) and ET-capacity (*ET)* was measured and the spare respiratory capacity (sc) was calculated (*P-ET)* in liver NCD or HFD (**A**) and the skeletal muscle of NCD or HFD (**B**) fed *Ptcd1*^+/+^ and *Ptcd1*^+/-^ mice using an OROBOROS oxygen electrode supplemented with succinate (S pathway control state) as the substrate in the presence of inhibitors and FCCP. FAO controlled pathway oxygen consumption was measured in the liver (**C**) and skeletal muscle (**D**) of NCD or HFD *Ptcd1*^+/+^ and *Ptcd1*^+/-^ mice using palmitoyl carnitine as the substrate in the presence of inhibitors to measure FAO Leak State (F_*L*_), FAO controlled OXPHOS Capacity (F_*P*_) FAO controlled OXPHOS capacity in the presence of a succinate (FS_P_) and FAO ET-capacity in the presence of succinate and FCCP (*FS_E_*). Data are from 5 mice from each genotype run in technical duplicates. Error bars indicated SEM. *P<0.05, Student’s t test.

We measured mitochondrial respiration in the liver and skeletal muscle of the young *Ptcd1^+/-^* mice for the N-pathway that delivers electrons from NADH to Complex I, and S-pathway that delivers electrons from succinate to Complexes II-III, where the Leak (*L*) is the rate of oxygen consumption in the absence of ADP or non-phosphorylating state 4/2, OXPHOS capacity (P) is the rate of oxygen consumption in the presence of ADP or phosphorylating state 3 and electron transport (ET)-Capacity (ET) state is the measure of maximal respiration or uncoupled respiration. We show that oxygen consumption was significantly reduced in the skeletal muscle in the Leak (*L*), OXPHOS (*P*)-capacity ET (*E)*-capacity states compared to control mice on a NCD and HFD (Figure 7A). There was no significant change observed in the liver of NCD or HFD (Figure 7A) *Ptcd1^+/-^* mice. However, the spare oxidative respiratory capacity (calculated as the difference between *P* and *ET*) of skeletal muscle mitochondria from the *Ptcd1^+/-^* mice fed a HFD was significantly reduced (Figure 7B) compared to the respiratory capacity in liver mitochondria that was not different between the haploinsufficient and control mice (Figure 7A). These findings suggest that liver mitochondria use the maximal respiratory capacity to increase fuel oxidation when there is increased lipid uptake enabling them to maintain mitochondrial biogenesis [35]. This was not possible in skeletal muscle mitochondria because the decreased spare capacity reduced the ability of the skeletal muscle to respond to an increased energy demand to compensate for the protein synthesis defect. Taken together our findings suggest that despite increased lipid availability the defect in mitochondrial protein synthesis cannot be compensated for in skeletal muscle because mitochondrial lipid uptake and spare respiratory capacity are reduced.

We investigated the effects of reduced mitochondrial protein synthesis with mice fed NCD and HFD on palmitate oxidation in the liver and skeletal muscle by measuring mitochondrial oxygen consumption rates (OCR) in isolated mitochondria using palmitoylcarnitine, that is an intermediate in mitochondrial fatty acid oxidation, as a substrate. In the livers of NCD *Ptcd1^+/-^* mice under the fatty acid oxidation pathway control state or F-pathway control, Leak respiration (F*L)* (the presence of reducing substrates, but absence of ADP) is significantly upregulated, along with the uncoupled state of the *ET-*capacity *(FS_E_*) (Figure 7C). In the HFD-fed *Ptcd1^+/-^* mice, palmitoycarnitine only contributes to increased respiration in the presence of succinate, whereas respiration is decreased in the presence of an uncoupler (Figure 7C). In the skeletal muscle of NCD Leak respiration (F*L)* along with OXPHOS capacity *(F_P_*) and the uncoupled rate are increased compared to their control littermates (Figure 7D), while in HFD *Ptcd1^+/-^* mice, Leak respiration (F*L)* along with OXPHOS capacity *(F_P_*) and ET-Capacity (*FS_P_*) remain similar to their control littermates. These data indicate that when mitochondrial protein synthesis is decreased, HFD can result in increased respiratory capacity in the liver by partial uncoupling.

## DISCUSSION

Recently we identified that reduction in PTCD1, a protein required for mitochondrial protein synthesis [16], can lead to adult-onset obesity [20]. Here we dissect the mechanisms that lead to adult-onset obesity by investigating how reduction in mitochondrial protein synthesis from early age in haploinsufficient *Ptcd1* mice can affect metabolism and cell signaling on normal and high fat diets. We identify that a high fat diet causes glucose tolerance and insulin sensitivity when mitochondrial protein synthesis is reduced, which protects the mice from weight gain at an early age. As a consequence, hormones, growth factors and cytokines involved in glucose, lipid metabolism, regulation of food intake and energy expenditure were not significantly affected, and reduced insulin levels in the serum of *Ptcd1^+/-^* mice were consistent with the lower overall body weight and increased insulin sensitivity and utilization. Although reduced mitochondrial protein synthesis appeared to protect from diet induced metabolic dysfunction and obesity, SCFA analyzes suggested that a decrease in the levels of butyrate and an increase in acetate was indicative of potential early onset metabolic reprogramming. Evidence in animal models has indicated that the gut microbiota affects host physiology and in particular muscle and WAT function [36, 37]. Consistent with this, we identified that reduction in mitochondrial protein synthesis causes inflammation in WAT and the skeletal muscle that are further exacerbated on the high fat diet leading to muscle and WAT fibrosis. In contrast, we identified reduced lipid accumulation in the livers of high fat fed *Ptcd1^+/-^* mice, suggesting that, despite activation of the SAPK/JNK pathway, the liver can be protected from diet induced steatosis. Activation of SAPK/JNK in the livers of high fat fed *Ptcd1^+/-^* mice likely reflects the upregulation of the stress response through the increase in CHOP levels. This signaling response has been identified previously [38] to enable cell survival in an effort to restore energy homeostasis via a mitochondrial dependent pathway [39–41] and may explain the improved health of the liver but not the muscle of HFD-fed *Ptcd1^+/-^* mice.

The tissue-specific cell signaling in response to reduced mitochondrial protein synthesis observed in the aged *Ptcd1^+/-^* mice where mTOR signaling was dampened in the livers of these mice and was significantly upregulated in the heart and skeletal muscle, reflects the differential energy requirements between highly proliferative and post-mitotic tissues [20]. In the livers of young mice, decreased mTOR signaling was more evident when the mice were fed a HFD that correlated with increased phosphorylation of Akt. Enhanced Akt signaling can increase insulin responsiveness which may account for the insulin sensitivity and glucose tolerance observed in high fat fed *Ptcd1^+/-^* mice. This is supported with evidence from studies using liver-specific *raptor* knockout mice that have increased glucose tolerance in response to enhanced Akt signaling [42, 43] and dominant-negative *raptor* mice that have improved insulin sensitivity in the liver [44]. Furthermore, an adipose-specific *raptor* knockout mouse was found to be lean and resistant to diet-induced obesity and have improved glucose tolerance and insulin sensitivity [45]. Reduced mTOR signaling in the liver of *Ptcd1^+/-^* mice may be a way to protect liver function against the development of insulin resistance and metabolic dysfunction early in life. In contrast, the heart and skeletal muscle have high energy demands and depend on OXPHOS, therefore decline in mitochondrial energy supply may trigger mTOR activation to generate more energy from the cytoplasm. This was evident with the increase in mTOR in the heart and skeletal muscle of normal diet fed *Ptcd1^+/-^* mice and no activation of mTOR signaling in these tissues with the high fat diet that provided additional nutrition for the energy demands of these tissues. Interestingly, terminal insulin stimulation indicated that insulin-dependent Akt activation is significantly reduced in the skeletal muscle of NCD mice, but not HFD mice, whereas it is elevated in the liver for both diets, providing further evidence that Akt activation protects the liver from insulin resistance, as previously observed [46].

Differential regulation of Akt signaling likely contributes to the tissue-specific responses of the liver and skeletal muscle to reduced mitochondrial protein synthesis. The HFD stimulated the increase in fatty acid import and metabolism in liver mitochondria that increased the biogenesis of mitochondrial OXPHOS proteins in the *Ptcd1^+/-^* mice. This was consistent with the reduced lipid content during a HFD and insulin sensitivity. In contrast, OXPHOS biogenesis was increased, although the uptake of fatty acids into mitochondria was greatly reduced in the skeletal muscle, possibly in response to reduced insulin signaling and Akt activation. Our findings support the requirement for Akt-stimulated insulin regulation of the CPT system within mitochondria, as has been observed before [47–49].

Spare respiratory capacity is essential for maintaining cellular function during stresses such as a HFD, with breaching of the maximal respiratory capacity leading to loss of the reserve capacity and organ dysfunction [50]. Increased requirements for ATP-linked oxygen consumption led to diminished reserve capacity in the *Ptcd1^+/-^* mice when mitochondrial protein synthesis is reduced. Therefore, increased ATP demand and decreased mitochondrial efficiency are likely primary drivers resulting in increased oxygen consumption and utilization of the reserve capacity in the liver. Glucose can lower maximal capacity due to compensatory increases in glycolysis and negative regulation of OXPHOS by glycolytic intermediates [51]. The Akt pathway has been shown to regulate reserve capacity [52], and it is likely that the liver is protected from steatosis and damage by increased use of the spare capacity in response to insulin-stimulated Akt upregulation. Mitochondrial reserve capacity is essential for skeletal muscle, affecting the metabolic state by accounting for ~80% of postprandial insulin stimulated glucose disposal [53] and is the main site of free fatty acid utilization. Therefore, the loss of the spare reserve capacity in skeletal muscle with the dampened Akt response predisposes the skeletal muscle to diet-induced fibrosis. Loss of mitochondrial reserve capacity in response to stress has also been observed in models of renal, cardiovascular and neurodegenerative diseases, as well as MERRF syndrome [54–56]. Glycolysis partially compensates for the loss or decrease of ATP production following mitochondrial dysfunction [55], however, if the reserve capacity is not sufficient to meet the additional energy demand in response to stress this can result in muscle pathologies including heart disease, and cell death in smooth muscle [50, 57, 58].

Here we show that defects associated with reduced mitochondrial protein synthesis can be compensated for in the liver by a HFD that can mobilize Akt-stimulated fatty acid oxidation and use of the respiratory spare capacity to rescue mitochondrial biogenesis and protect from steatosis. However, HFD can only partially protect from mitochondrial protein synthesis defects in skeletal muscle and WAT, as this defect selectively impairs Akt-mediated insulin signaling pathways, suggesting that upregulation of Akt in these tissues may be a possible target for treatments of mitochondrial dysfunction.

## MATERIALS AND METHODS

### Animals, feeding and housing

*Ptcd1* transgenic mice on a C56NL/6N background were generated as previously reported [20] by the Australian Phenomics Network (APN; Monash University, Melbourne, Australia). Male age- and litter-mate matched wild-type (*Ptcd1^+/+^*) and heterozygous (*Ptcd1^+/-^*) mice were housed in standard cages (45 cm x 29 cm x 12 cm) under a 12-hour light/dark schedule (lights on 7 a.m. to 7 p.m.) in controlled environmental conditions of 22± 2°C and 50 + 10% relative humidity. Mice were fed either a normal chow diet (Rat and Mouse Chow, Specialty Feeds, Perth, Australia) or a high fat diet (19 MJ/kg, 35% of energy from carbohydrate, 42% from fat, 23% from protein; Specialty Feeds, Perth, Australia) from five weeks of age and monitored for 12 weeks. Food and water were available *ad libitum*, with the exception of a five hour fast prior to metabolic experiments. This study was approved by the Animal Ethics Committee of the UWA and performed in accordance with Principles of Laboratory Care from the NHMRC Australian code for the care and use of animals for scientific purposes, 8^th^ Edition 2013.

### Tissue homogenate preparation

Tissue homogenates were prepared from 3 mm x 3 mm tissues pieces (liver, heart and skeletal muscle) and homogenized using a bead beater in 150 μl of Cell Extraction Buffer containing PhosSTOP Phosphatase Inhibitor Cocktail (Roche) and EDTA-free Complete protease inhibitor cocktail (Roche), as previously described [20]. The tissue homogenate protein concentration was quantified using the bicinchoninic acid (BCA) assay.

### Mitochondrial isolation

Mitochondria were collected from homogenized hearts and livers as previously described [14, 20, 59] and isolated by differential centrifugation as described previously [60], with some modifications. Skeletal muscles were collected in 2% collagenase (Sigma) and homogenized in buffer containing 210 mM mannitol, 70 mM sucrose, 10 mM Tris, 0.1 mM EDTA pH 7.4 containing EDTA-free Complete protease inhibitor cocktail (Roche) before differential centrifugation. The mitochondrial protein concentration was quantified using the Bicinchoninic acid (BCA) assay.

### Immunoblotting

Specific proteins were detected using the following antibodies in Odyssey blocking buffer (LI-COR Biosciences). **Rabbit monoclonal antibodies**: Phospho-mTOR (Ser^2448^, 5535), mTOR (2983), Phospho-SAPK/JNK(Thr^183^/Tyr^185^, 4668), SAPK/JNK (9252), Phospho-AMPKα (Thr^172^, 2531), Phospho-Akt (Ser^473^, 9272), Akt (9272), Phospho-S6 (Ser^235/236^, 4856), S6 (2217), Phospho-4E-BP-1 (Thr^37/36^, 2855), 4E-BP-1 (9644), Rictor (2140), ACC (3662), GAPDH (2118), Cell Signaling Technologies, diluted 1:500. **Rabbit polyclonal antibodies:** ACACA (pACC Ser^79^, OAAN02936) and CPTII (OAAN00972), Aviva Systems Biology, diluted 1:500, OXA1-L (21055-1-AP), Proteintech, diluted 1:1000. **Mouse monoclonal antibodies:** Total OXPHOS Cocktail Antibody (ab110412), and VDAC (ab14734), Abcam, diluted 1:1000. IRDye 800CW goat anti-rabbit immunoglobulin G (IgG) or IRDye 680LT goat-anti-mouse IgG (LI-COR Biosciences) secondary antibodies were used and the immunoblots where visualized using an Odyssey infrared imaging system (LI-COR Biosciences). Protein Densitometry was determined using ImageJ software.

### RNA isolation and qRT-PCR

RNA was isolated from total liver or skeletal muscle using the miRNeasy Mini kit (Qiagen) incorporating an on-column RNase-free DNase digestion to remove all DNA. Complementary DNA (cDNA) was prepared using the QuantiTect Reverse Transcription Kit (Qiagen) and used as a template in the subsequent PCR that was performed using a Corbett Rotorgene 6000 using SensiMix SYBR mix (Bioline) and normalized to 18S rRNA.

### Translation assays

*In organello* translation assays were carried out in isolated liver and skeletal muscle mitochondria as described before [14, 16]. Briefly, 500 μg mitochondria were incubated in 750 μl translation buffer (100 mM mannitol, 10 mM sodium succinate, 80 mM KCl, 5 mM MgCl_2_, 1 mM KPi, 25 mM HEPES pH 7.4, 5 mM ATP, 20 μM GTP, 6 mM creatine phosphate, 60 μg/ml creatine kinase and 60 μg/ml of all amino acids except methionine and cysteine). Mitochondria were supplemented with 150 μCi of ^35^S methionine and cysteine (PerkinElmer) for 60 min at 37^°^C. For chase experiments, after labeling, mitochondria were washed three times and incubated for 1 h at 37°C in translation buffer including cysteine and methionine. After labeling or chase, mitochondria were washed in translation buffer and suspended in 1% Triton X-100. Protein concentration was measured and 50 μg for liver and 25 μg for skeletal muscle of mitochondrial protein was resolved by SDS-PAGE and visualized by autoradiography.

### Oxygen consumption measurements

Mitochondrial respiration was evaluated as O_2_ consumption in isolated liver and skeletal muscle mitochondria, as previously described [14, 16]. Mitochondria were supplemented with 10 mM succinate / 0.5 μM rotenone (Sigma), to measure ADP-independent respiration activity (Leak State (*L))*). After addition of saturating ADP (Sigma), OXPHOS capacity (*P)* was measured. Respiration was uncoupled by successive addition of FCCP up to 3 μM to reach maximal respiration (ET-capacity (*ET*)). Spare respiratory capacity was calculated from the difference between OXPHOS capacity and ET-capacity (*sc=P-ET)*.

Fatty acid oxidation was measured from isolated liver and skeletal muscle mitochondria from both normal control and high fat diet mice that were fasted for 5 hours prior to sacrifice. Using the Oxygraph 2K respirometer (Oroboros Oxygraph-2K, Oroboros Instruments Corp, Innsbruck, Austria) as described by [61], and reviewed by [62]. Respiration supported by palmitoylcarnitine (Pal) was determined under Leak state in the presence of 0.1 mM malate (Sigma) and 1 mM palmitoylcarnitine (Sigma), OXPHOS capacity, was measured using, 1 mM K-ADP (Sigma) and 10 mM K-succinate was added to measure ET-capacity (Sigma) Inhibitors used included 0.5 μM oligomycin (Sigma) and 2.5 μM antimycin. Carbonyl cyanide p-trifluoro-methoxyphenyl hydrazone (FCCP) was used as a measure of uncoupling and maximal respiration rate in titrations up to 3 μM.

### Histochemistry

Fresh sections of the liver, skeletal muscle, and white adipose tissue were frozen in optimal cutting temperature (OCT) medium or fixed in 10% Neutral buffered formalin and then embedded in paraffin wax, sectioned in 5 μm to 10 μm sections and stained with haematoxylin and eosin or Oil Red O and haematoxylin. Images were acquired using a Nikon Ti Eclipse inverted microscope using a Nikon 20x objective and staining was quantified as described previously [20, 59].

### Metabolic assays

An intraperitoneal glucose tolerance test (GTT) and insulin tolerance test (ITT) were performed on high fat diet mice as described previously [20, 63]. Mice were fasted for five hours at weeks 5 and 10, and 6 and 11 for GTT and ITT, respectively. Blood samples were obtained from the tail tip at the indicated times and glucose levels were measured using a glucometer (AccuCheck II; Roche. The doses used during these tests were 1 g/kg body weight and 0.5 U/kg body weight for GTT and ITT, respectively. A subgroup of NCD and HFD mice received terminal insulin injections of 1 U/kg body weight for 5 minutes prior to being culled and key metabolic tissues were collected to determine insulin responsiveness. Cardiac blood samples were taken and measured for insulin (Merck Millipore), leptin, adiponectin, fibroblast growth factor 21 (R&D Systems) and IL-6 (Jomar Life Science) using standard ELISA kits according to manufacturer's instructions. Tissue IL-6 levels from muscle, liver and white adipose tissue were measured as described previously [64]. Data were analyzed using an online software program (https://elisaanalysis.com/) and the area under the curve was calculated using the trapezoidal rule using excel software (Microsoft, 2007). SCFA profiling was performed as described previously to measure the concentration of acetic acid or butyric acid as nanomoles per microlitre of serum [65]. Serum triglyceride levels were measured by Pathwest Laboratory Medicine (Nedlands, WA, Australia). Tissue triglyceride levels were quantified using the Abcam Triglyceride Assay Kit (ab65336). Briefly, 100 mg of tissue was homogenized in 5% NP-40/ddH_2_O and slowly heated to 100°C in a water bath until the solution became cloudy, then cooled and repeated to solubilize the triglycerides. The samples were then centrifuged for 2 minutes at maximum speed to remove insoluble material. Liver samples were diluted to a working concentration of 20-fold and skeletal muscle samples were diluted 10-fold with ddH_2_O. The colorimetric triglyceride quantification was performed according to the manufactures instructions and measured at OD 570 nm using a microplate reader. All data were statistically evaluated by a two-tailed Student’s *t* test and a one-way or two-way ANOVA at a significance level of *p* < 0.05 using excel (Microsoft, 2007) and StatPlus (AnalystSoft, v.5) software.

## Supplementary Material

Supplementary Figures
