# Supplementary material for: Reduced mitochondrial translation prevents diet-induced metabolic dysfunction but not inflammation
Source: Aging (Albany NY). 2020 Oct 6;12(19):19677–700. doi: 10.18632/aging.104010 (PMC7732297; doi:10.18632/aging.104010)
Supplement: Supplementary Figures [file aging-12-104010-s001..pdf]

SUPPLEMENTARY FIGURES

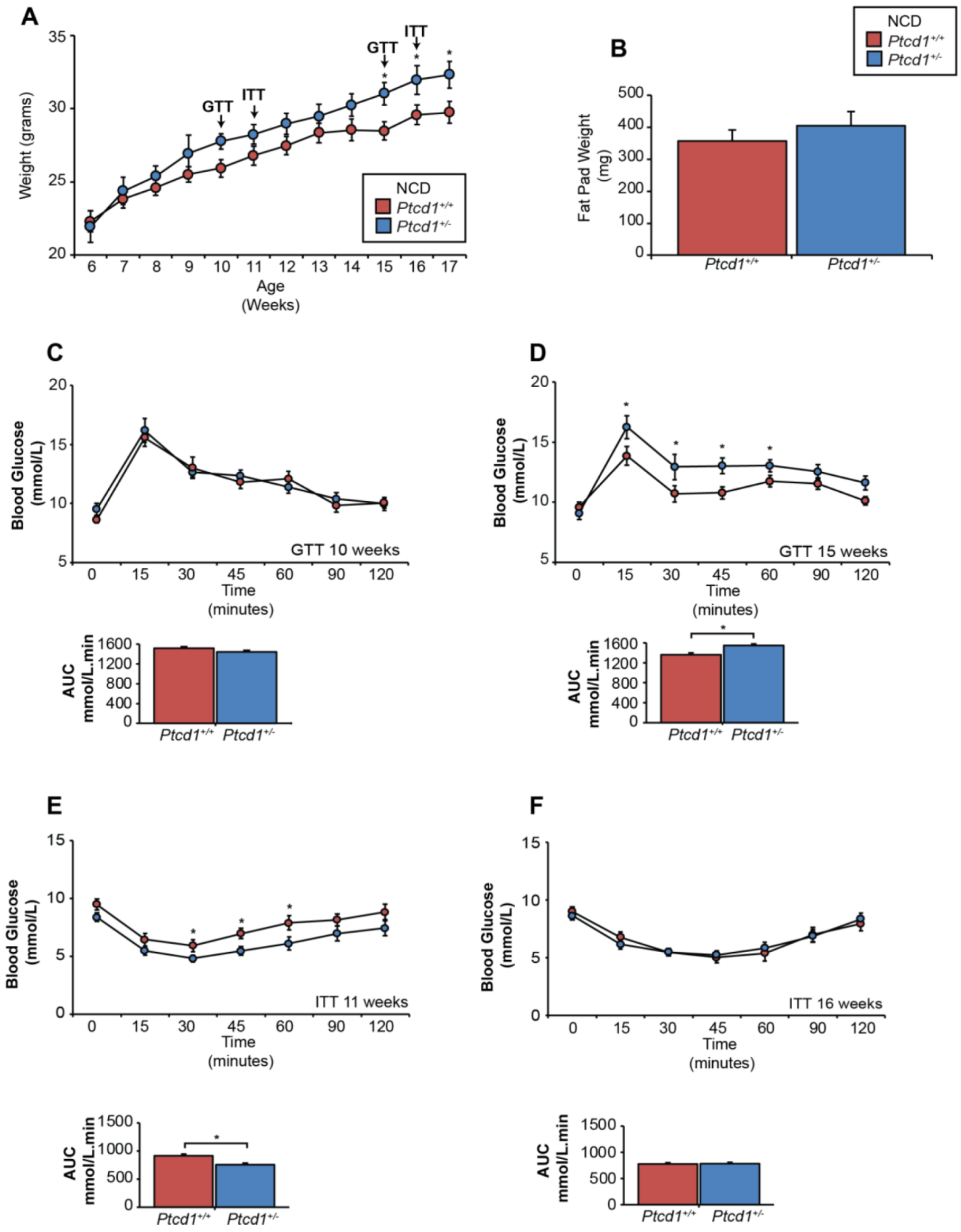

Supplementary Figure 1. Related to figure 1. The effects of normal chow diet on mice with reduced mitochondrial protein synthesis. (**A**) Weight progression in grams from 6 to 17 weeks of age between *Ptcd1*$^{+/+}$ (n=12) and *Ptcd1*$^{+/–}$ (n=12) mice fed a normal chow diet. (**B**) Weight of intra-abdominal epididymal fat pads in grams for NCD 17-week old Ptcd1$^{+/+}$ (n=12) and *Ptcd1*$^{+/–}$ (n=12) mice. Glucose tolerance in 10- (**C**) and 15-week-old (**D**) *Ptcd1*$^{+/+}$ (n=12) and *Ptcd1*$^{+/–}$ (n=12) mice. Insulin sensitivity in 11- (**E**) and 16-week-old (**F**) *Ptcd1*$^{+/+}$ (n=12) and *Ptcd1*$^{+/–}$ (n=12) mice. Quantitative values are the area under the curve (AUC) ± SEM. *$P < 0.05$, Student's *t* test.

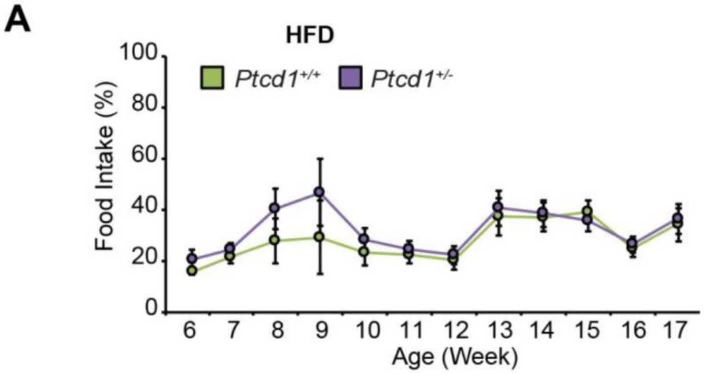

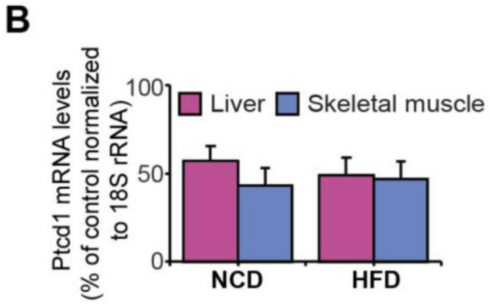

Supplementary Figure 2. Related to figure 1. The effects of reduced mitochondrial protein synthesis on food intake and *Ptcd1* expression. (**A**) Percentage of food intake from 12 weeks of HFD feeding between *Ptcd1*$^{+/+}$ (n=10) and *Ptcd1*$^{+/-}$ (n=10) mice. (**B**) Percent reduction in *Ptcd1* expression in liver and skeletal muscle from NCD or HFD fed *Ptcd1*$^{+/-}$ (n=5) mice relative to *Ptcd1*$^{+/+}$ (n=5) determined by qRT-PCR and normalized to 18S rRNA expression.

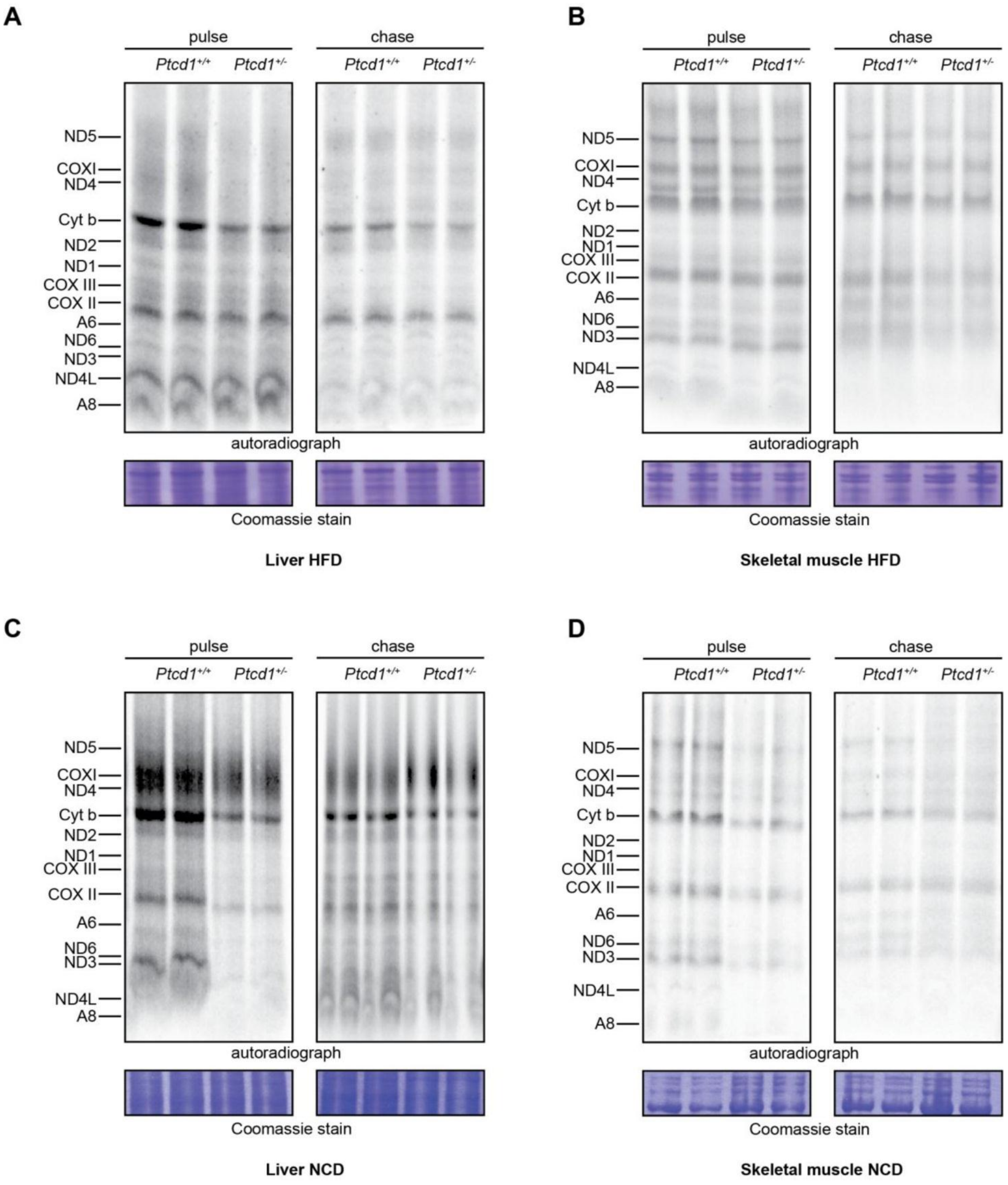

Supplementary Figure 3. The effects of diet on mitochondrial protein synthesis in *Ptcd1*$^{+/+}$ and *Ptcd1*$^{+/-}$ mice. *De novo* protein synthesis in mitochondria isolated from livers - and skeletal muscle - of young *Ptcd1*$^{+/+}$ and *Ptcd1*$^{+/-}$ fed either a high fat diet (**A**, **B**) or normal chow diet (**C**, **D**) was measured by pulse and chase incorporation of ^{35}S-labeled methionine and cysteine. Mitochondrial proteins were separated by SDS–PAGE, stained using Coomassie Brilliant Blue to show equal loading, and visualized by autoradiography.

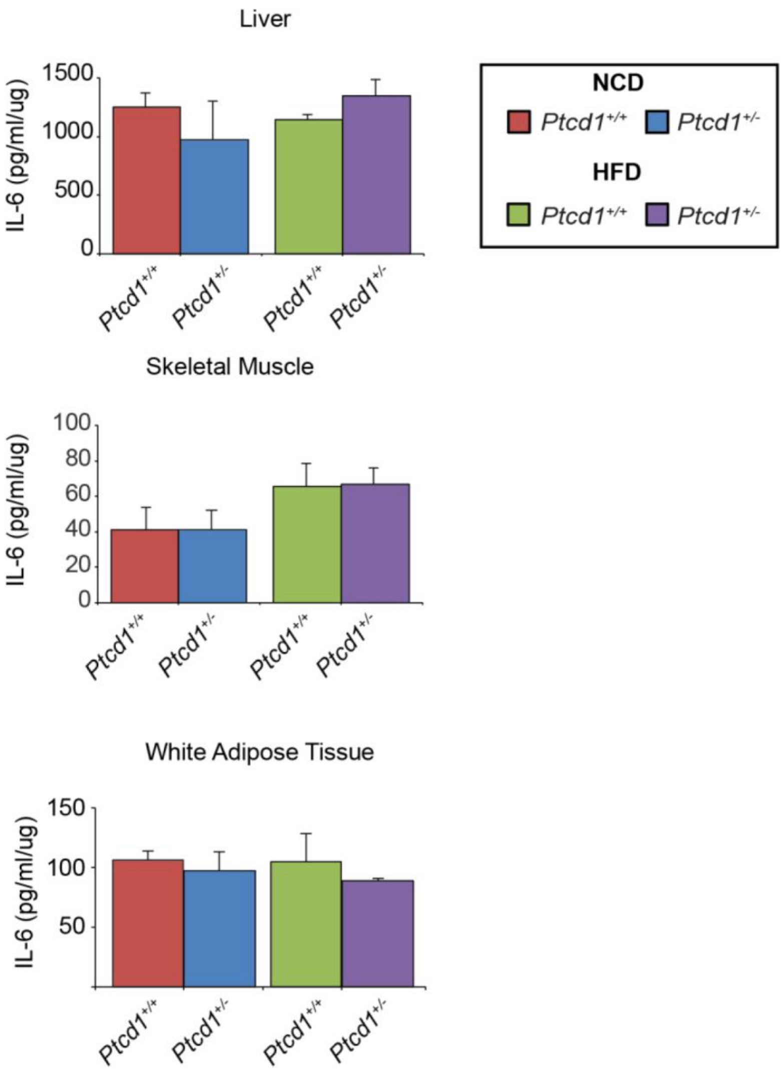

Supplementary Figure 4. Related to figure 2. The effects of reduced mitochondrial protein synthesis on IL-6 levels. IL-6 levels were measured in liver, skeletal muscle and white adipose tissue from *Ptcd1*$^{+/+}$ (n=5) and *Ptcd1*$^{+/-}$ (n=5) mice.

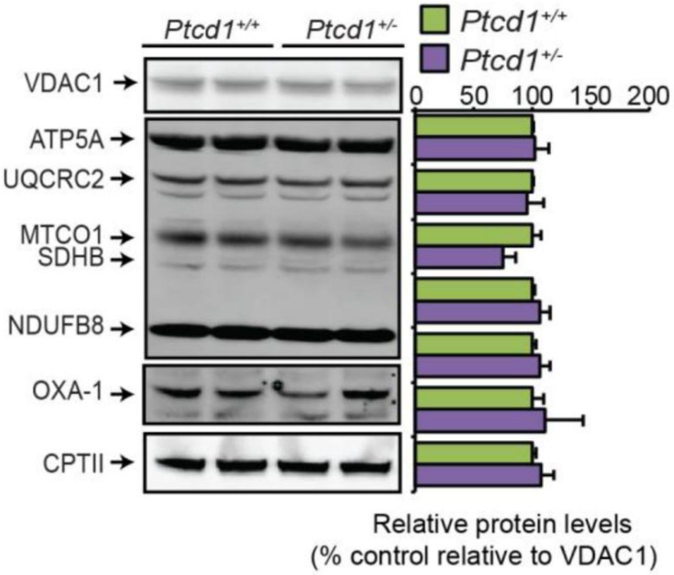

Supplemenatary Figure 5. Related to figure 6. The effects of HFD on heart mitochondrial biogenesis in *Ptcd1*$^{+/+}$ and *Ptcd1*$^{+/-}$ mice. Heart mitochondrial proteins (50 µg) isolated from *Ptcd1*$^{+/+}$ (n=5) and *Ptcd1*$^{+/-}$ (n=5), 17-week-old mice fed a high fat diet were resolved on 4-20% SDS-PAGE gels and immunoblotted against the OXPHOS antibodies to investigate the steady state levels of nuclear and mitochondrial encoded proteins. VDAC1 (porin) was used as a loading control. Relative abundance of the levels of the steady state proteins were calculated as a percentage compared to VDAC1. Results were analyzed using the Student's t test.
